# Rational use of antibiotics by community health workers and caregivers for children with suspected pneumonia in Zambia: a cross-sectional mixed methods study

**DOI:** 10.1186/s12889-016-3541-8

**Published:** 2016-08-27

**Authors:** Kirstie Graham, Chomba Sinyangwe, Sarala Nicholas, Rebecca King, Samuel Mukupa, Karin Källander, Helen Counihan, Mark Montague, James Tibenderana, Prudence Hamade

**Affiliations:** 1 Malaria Consortium, Development House, 56-64 Leonard Street, London, EC2A 4LT UK; 2 Malaria Consortium, Mansa, Luapula Province Zambia; 3 Nuffield Centre for International Health and Development, University of Leeds, Leeds, UK; 4 Malaria Consortium, Kampala, Uganda

**Keywords:** Pneumonia, Community health workers, Integrated community case management, Rational use, Antibiotics, Adherence, Caregivers, COMDIS-HSD, Malaria Consortium

## Abstract

**Background:**

Antibiotic resistance is an issue of growing global concern. One key strategy to minimise further development of resistance is the rational use of antibiotics, by providers and patients alike. Through integrated community case management (iCCM), children diagnosed with suspected pneumonia are treated with antibiotics; one component of an essential package to reduce child mortality and increase access to health care for remote populations. Through the use of clinical algorithms, supportive supervision and training, iCCM also offers the opportunity to improve the rational use of antibiotics and limit the spread of resistance in resource-poor contexts. This study provides evidence on antibiotic use by community health workers (CHWs) and caregivers to inform iCCM programmes, safeguarding current treatments whilst maximising access to care.

**Methods:**

1497 CHW consultations were directly observed by non-clinical researchers, with measurement of respiratory rate by CHWs recorded by video. Videos were used to conduct a retrospective reference standard assessment of respiratory rate by experts. Fifty-five caregivers whose children were prescribed a 5-day course of antibiotics for suspected pneumonia were followed up on day six to assess adherence through structured interviews and pill counts. Six focus group discussions and nine in depth interviews were conducted with CHWs and caregivers to supplement quantitative findings.

**Results:**

The findings indicate that CHWs adhered to treatment guidelines for 92 % of children seen, prescribing treatment corresponding to their assessment. However, only 65 % of antibiotics prescribed were given for children with experts’ confirmed fast breathing pneumonia. Qualitative data indicates that CHWs have a good understanding of pneumonia diagnosis, and although caregivers sometimes applied pressure to receive drugs, CHWs stated that treatment decisions were not influenced. 46 % of caregivers were fully adherent and gave their child the full 5-day course of dispersible amoxicillin. If caregivers who gave treatment for 3 to 5 days were considered, adherence increased to 76 %.

**Conclusions:**

CHWs are capable of prescribing treatment corresponding to their assessment of respiratory rate. However, rational use of antibiotics could be strengthened through improved respiratory rate assessment, and better diagnostic tools. Furthermore, a shorter course of dispersible amoxicillin could potentially improve caregiver adherence, reducing risk of resistance and cost.

## Background

In recent years, there has been increasing attention given to the issue of antibiotic resistance, as demonstrated by the World Health Organization (WHO) report on antimicrobial resistance and surveillance [1], and the global action plan on antimicrobial resistance [2], which was endorsed at the World Health Assembly in May 2015. The WHO report called for ‘concerted cross-sectional action by governments and society as a whole’ and described the issue as a ‘global health security threat’ [1]. With limited numbers of antibiotics available, and resistance to first-line treatments becoming increasingly common, resistance results in higher health care costs, as patients require more expensive and longer treatments, and a greater risk of mortality from diseases that were previously curable. In 2011, WHO highlighted a six-point policy package to combat the problem, including regulating and promoting the rational use of medicines [3].

The rational use of antibiotics not only concerns the actions of providers, in ensuring patients receive appropriate treatment for their condition, at the right dose and duration, but also those of patients, in adhering to the treatment regimens prescribed, completing the full course and not sharing or storing medicines for future use. In 2001, the WHO Global Strategy for containment of antimicrobial resistance [4] highlighted the need for the ‘development and use of guidelines and treatment algorithms to foster appropriate use of antimicrobials’, as well as the importance of ‘supervision and support of clinical practices, especially diagnostic and treatment strategies’. Furthermore, it highlighted that health care providers have an important role to play in educating patients on the importance of treatment adherence [4].

In many resource-poor contexts, with limited surveillance and weaker regulatory systems [5, 6], containment of resistance may be more challenging than in higher-income settings. However, in these settings, there remains a high burden of childhood infectious diseases, such as pneumonia, that often require antibiotic treatment. Pneumonia is responsible for the second largest number of child deaths globally, with a disproportionate burden and the highest number of deaths occurring in sub-Saharan Africa [7, 8]. Frequently, community members obtain antibiotics of unknown quality, without a prescription from local pharmacies, informal drug vendors, or private facilities, which are typically difficult to regulate and where prescription of medication is inherently linked to payment received [5, 9]. Integrated community case management (iCCM), a package of essential care delivered by community health workers (CHWs) for treatment of three key childhood diseases; pneumonia, malaria and diarrhoea, and often also the detection of malnutrition, aims to reduce childhood mortality and increase access to care for hard-to-reach populations. It also has the potential to encourage the rational use of antibiotics at community level and control the development or spread of antibiotic resistance through training, the use of simple job aids with effective clinical algorithms, diagnostic tools, availability of blister packaged (and sometimes colour coded) appropriate antibiotics, and supportive supervision, with CHWs acting as gatekeepers to these essential medicines. Furthermore, CHWs may be able to encourage and counsel caregivers to adhere to correct treatment regimens, for example, giving instructions regarding the number of doses to be given per day and the number of tablets per dose.

However, there remains a perceived risk that CHWs distributing antibiotics may add to selective pressure among pathogens [10], i.e. the misuse of antibiotics selectively encourages the growth of antibiotic resistant bacteria, and thus more evidence is needed surrounding the use of antibiotics at community level and specifically relating to iCCM. This study sought to provide evidence on the use of antibiotics by CHWs and caregivers for the treatment of suspected pneumonia in children under five to inform future implementation of iCCM programmes to help preserve the effectiveness of current antibiotics, whilst at the same time continuing to provide access to care for remote and marginalised populations [6]. This paper presents quantitative data on CHWs’ adherence to guidelines in prescribing antibiotics, the rational use of antibiotics including dosage prescribed, and caregivers’ adherence in administering the course of treatment prescribed. In addition, qualitative data helps to provide context, exploring some of the reasons that may influence adherence by both populations respectively, for example, community perceptions on CHW prescribing practices, and caregivers’ expectation of care provided.

## Methods

### Study setting

Data collection was conducted in Samfya and Kawambwa districts of Luapula province in northern Zambia between October and December 2012. The two study districts are situated in the south and the north of the province, respectively, with populations of 191,980 and 130,680 [11], and are representative of the other districts in the province. iCCM was implemented in Luapula province from 2009 to 2013, funded by the Canadian International Development Agency (CIDA), with a total of 440 CHWs trained in iCCM in these two districts. In this context, CHWs are typically non-clinical unpaid community volunteers, who have received little formal training. At a minimum, all CHWs involved in this study had received the 6-day iCCM training supported by Malaria Consortium, as part of the iCCM programme.

### Study design

Nine field researchers observed 1497 CHW consultations conducted by 90 CHWs, in CHWs’ usual work context (at their own or their patient’s home), using a structured checklist. For the 698 children with suspected pneumonia, as assessed by the CHW, the field researcher filmed the CHW measuring the respiratory rate of the child. Every field researcher visited ten CHWs for 3 days each. On the first day of each 3 day visit, the community was sensitised to encourage them to bring their sick children to the CHW during the next 2 days. Subsequently, the CHW’s consultations were observed for these 2 days. The video recordings were reviewed retrospectively by two pairs of a total of four experts to obtain a reference standard assessment of respiratory rate. Experts were national-level certified master trainers in Integrated Management of Childhood Illnesses (IMCI) in Zambia. Data comparing the expert and CHW assessment of fast breathing, and the proportion of children appropriately treated has been reported separately [12], as the focus of this manuscript is primarily on the rational use of antibiotics in this context, rather than the quality of care received. However, in summary, the data support existing literature that CHWs are capable of measuring respiratory rates and providing appropriate treatment for suspected pneumonia.

Following each observation period, the field researchers conducted follow-up home visits with caregivers whose children had been previously prescribed antibiotics by the 90 CHWs involved in the observation component of the study. The antibiotics prescribed were a 5-day course of dispersible amoxicillin in age-specific and colour-coded blister packs. Caregivers were identified from CHW records. A total of 55 caregiver interviews were conducted; 42 interviews (76 %) in Samfya district, and 13 in Kawambwa district. During each home visit, field researchers conducted a pill count of the tablets remaining in the pack, and a structured interview to collect a self-report of caregiver adherence.

To further elaborate on the quantitative findings, six focus group discussions (FGD) with CHWs and caregivers, and nine in depth interviews (IDI) with CHWs were conducted. All qualitative study components were audio-recorded and conducted in the local language, Bemba. The data were subsequently translated and transcribed into English, either verbatim for the IDIs, or using the Fairnotes approach [13] for the FGDs. IDIs were conducted at the end of the 2 days of observation with the CHW to be interviewed, and FGDs were conducted once the observation of all 90 CHWs was completed. The IDIs took approximately 40 min to conduct, whilst the FGDs were about 2 h in length.

The field researchers were all Zambian nationals from Luapula province with no clinical experience. The field researchers participated in 7 days of interactive training on all the study components, including detailed review of the question guides to ensure shared understanding of the translated material.

### Participants and sampling

The sample size for the observation component of the study was 90 CHWs, calculated to identify the proportion of CHWs who adhere to iCCM guidelines. This assumed 50 % of CHWs adhered to iCCM guidelines, at a precision of 10 %, within a finite population of 440 CHWs that were trained in iCCM in the two study districts and included an adjustment of 10 % to account for CHW non-response and data excluded from final analysis for any reason. The findings presented on correct use of antibiotics are a result of secondary analysis of the data collected. CHWs were selected for inclusion in the observation component of the study by simple random sampling, from the total number of CHWs trained in iCCM in the two study districts.

The sample size for the follow-up visits to assess caregiver adherence was calculated to identify the proportion of caregivers who adhere to the antibiotic treatment regimen. The total number of caregivers required was 132, assuming 50 % adherence to antibiotic regimens, at a precision of 10 %, an adjustment for clustering of caregivers by CHW, with a design effect of 1.25. Caregivers of all simple pneumonia cases prescribed antibiotics 6 days prior to the day when data collection was done were eligible for inclusion in this study component. Unfortunately, due to logistical issues relating to the amount of distance to be travelled by each field researcher, it was not possible to interview all who were eligible and reach the required sample size. A total of 55 caregivers were interviewed. Whilst this is a limitation of the data presented, the data remains of value given the limited evidence available on antibiotic adherence by patients in low-income country contexts.

For the IDIs, nine CHWs were systematically sampled, with the eighth CHW observed by each of the nine field researchers invited to participate at the end of the second day of observation. For the six FGDs, three of which were conducted with CHWs, and three with caregivers, 24 CHWs and 24 caregivers were selected by convenience sampling. All CHWs and caregivers who had participated in the observations and day six follow-up interviews, respectively, were eligible to participate, as well as other caregivers whose children had recently received treatment for suspected pneumonia from the CHW.

### Analysis

Quantitative data were analysed using Stata version 12 (StataCorp LP). Of the 1497 CHW consultations observed, seven were excluded due to an absence of data being recorded (*n* = 2), the child being older than 5 years of age (*n* = 1) or the age of the child not specified (*n* = 4). Subsequently, of the 698 videos recorded of the respiratory rate count conducted by the CHW during these consultations, 161 videos were excluded due to poor video quality, missing information or if the child was breastfeeding or crying or where the expert ratings of respiratory rate differed by more than five counts and were not recounted. Therefore, data from 1490 consultations and 537 videos were analysed to assess CHW adherence to guidelines and antibiotic use. Videos were retrospectively reviewed by the experts, assessing CHWs’ counts against a reference standard.

Descriptive results are summarised as frequencies, percentages or as a median with inter-quartile range [IQR]. A number of indicators were calculated to assess the percentage of children with fast and normal breathing, as classified by the CHW, who were treated in line with the iCCM guidelines. Indicators were also developed to determine whether antibiotics were given for the correct purpose i.e. fast breathing pneumonia as assessed by an expert, and at the correct dosage.

Each indicator is presented as a percentage with 95 % confidence intervals adjusting for clustering of children by CHW. Pearson chi-squared test accounting for clustering was used to assess association between each indicator and various factors (e.g. younger and older children).

Data from all 55 caregiver interviews was analysed to explore caregiver adherence to antibiotic treatment. Adherence was measured using the pill count of remaining antibiotics and a self-report on the frequency of giving antibiotics to the child. From these, a combined indicator of adherence was created. Full adherence was defined as a caregiver who reported giving the child antibiotics three times a day, at the correct times (morning, noon and night) for 5 days and who had no tablets remaining in the pack. In all other scenarios, caregivers were classified as non-adherent. Results are presented as summary statistics (means or percentages). The percentage adherent is presented with confidence intervals, accounting for the cluster effect.

Qualitative data was managed using NVivo 10 (QSR International) and analysed by thematic analysis. The coding frame was developed by three researchers (RK, KG, CS) based on analysis and discussion of three CHW IDI transcripts. It consisted of both a priori and grounded codes. The remaining IDI and FGD transcripts were analysed by KG, with further discussion as needed regarding any changes to the coding frame. Summaries of the qualitative data were reviewed by CS and RK.

## Results and discussion

### Participants

As presented in Table 1, approximately 80 % of the 90 CHWs who were observed were male, 77 % had a secondary school education, and 90 % originated from the community in which they worked. The CHWs had a wide range of experience, with 23 % having worked for longer than 10 years, 21 % between 5 to 10 years and 53 % less than 5 years. The median number of children observed per CHW was 14 [IQR: 10–21] during the two days observation period. Half the children were female and 25 % were aged 2–11 months. The characteristics of the children whose respiratory rate was recorded on video were similar to those whose respiratory rate was not counted.

**Table 1:** CHW characteristics

	CHW	
	*N* = 90	
Median Age, [IQR]	44	[38,49]
Sex, n(%)		
Female	17	(19)
Highest level of education, n(%)		
Primary	19	(21)
Secondary	69	(77)
College	1	(1)
Not specified	1	(1)
Originate from community where they work, n(%)	81	(90)
Years working as CHW, n(%)		
< 1	1	(1)
1 to 2	22	(24)
2 to 5	26	(29)
5 to 10	19	(21)
> 10	21	(23)
Not specified	1	(1)

Of the 55 caregivers interviewed, 93 % were mothers and the remaining 7 % were close family members (Table 2). Almost half of the caregivers were above 30 years of age and the majority (82 %) had a primary school education. The age of the sick child was recorded by field researchers in 42 interviews and the age ranged from 3 months to 4 years, with 38 % in the younger age category (2–11 months).

**Table 2:** Caregiver characteristics

Caregiver characteristics	Mothers *N* = 51	Others *N* = 4	Total *N* = 55
Age of caregiver (years)			
< 30	29 (57 %)	0 (0 %)	29 (53 %)
> = 30	22 (43 %)	4 (100 %)	26 (47 %)
Highest Education			
None	3 (6 %)	1 (25 %)	4 (7 %)
Primary	42 (82 %)	3 (75 %)	45 (82 %)
Secondary or above	6 (12 %)	0 (0 %)	6 (11 %)
Age of child			
2–11 months	16 (31 %)	0 (0 %)	16 (29 %)
12–59 months	24 (47 %)	2 (50 %)	26 (47 %)
Not specified	11 (22 %)	2 (50 %)	13 (24 %)

The majority of CHWs who participated in the IDIs were male. The CHW FGDs involved 17 males and seven females, with eight participants per group. All 24 participants in the caregiver FGDs were female, and brought children with them.

### Rational use of antibiotics by CHWs

Results are reported for the 537 observations with a video recording, where it was possible for experts to verify the respiratory rate count determined by the CHW.

### Adherence to treatment guidelines

Of all the children whose respiratory rate was counted by the CHW, 92 % [95 % CI: 87, 95] were treated according to the treatment guidelines, receiving antibiotics for fast breathing and no antibiotics for a normal respiratory rate (Table 3). There was no significant difference in CHW adherence to the guidelines for prescribing antibiotics between the two age groups (*p* = 0.31).

**Table 3:** Medication prescribed by CHW according to assessment of fast breathing by CHW and child’s age

	All children *N* = 537		Children aged 2–11 months *N* = 147		Children aged 12–59 months *N* = 390	
	Normal breathing (%)	Fast breathing (%)	Normal breathing (%)	Fast breathing (%)	Normal breathing (%)	Fast breathing (%)
	*N* = 324	*N* = 213	*N* = 68	*N* = 79	*N* = 256	*N* = 134
Received Antibiotics, n						
No	308 (95)	27 (13)	65 (89)	6 (8)	243 (95)	21 (16)
Yes	16 (5)	186 (87)	3 (4)	73 (92)	13 (5)	113 (84)
Colour of Pack Given, n						
Pink (for children aged 2–11 months)	7 (44)	78 (41)	2 (67)	66 (90)	5 (38)	12 (11)
Green (for children aged 12–59 months)	8 (50)	99 (53)	0 (0)	3 (4)	8 (62)	96 (85)
NS^a^	1 (6)	9 (5)	1 (33)	4 (6)	0 (0)	5 (4)
% received antibiotics [95 % CI]	5 [2,10]	87 [78,93]	4 [1,14]	92 [80,97]	5 [2,12]	84 [75,91]
*p*-value*		<0.01		<0.01		<0.01

*P-value comparing proportion receiving antibiotics by fast breathing status (CHW), for all children and by age group

^a^ *NS* not specified

CHWs’ ability to prescribe antibiotics according to the guidelines and corresponding to their assessment suggests that CHWs are capable of treating pneumonia at community level. Overall, as shown in Table 3, 87 % [95 % CI: 78, 93] of children with fast breathing, as assessed by the CHW, were prescribed antibiotics. The difference between the number receiving antibiotics for fast breathing in the two age groups was not significant (*p* = 0.06). These data are in line with studies by Mukanga et al. [14] and Kalyango et al. [15], with the latter stating that 82 % of children with fast breathing had received an antibiotic. To note, 6/27 of the children with fast breathing who were not given amoxicillin were referred. Despite these children not being prescribed antibiotics, they may still have been appropriately managed by the CHW, for example if the CHW lacked the appropriate medication.

The proportion receiving antibiotics was significantly lower among those with normal breathing (*p* < 0.01), in the two age groups, when analysed separately, and for all children (Table 3).

When the data was further analysed to take into account the dosage of antibiotics given, 79 % [95 % CI: 70, 87] of the children with fast breathing as assessed by the CHW were prescribed antibiotics and at the correct dose for the child’s age. A significantly higher proportion of children in the younger age group received antibiotics for fast breathing and at the correct dose compared to older age group (*p* = 0.02).

Of all the children prescribed antibiotics by CHWs, regardless of whether or not the child had fast breathing, 10 % received antibiotics that were at the incorrect dose for the child’s age (Table 3). 14 % of the older children received the pink pack of antibiotics intended for the younger children, whilst 4 % of the younger children received the green pack of antibiotics intended for the older children. The pack given was not specified for ten children, five in the younger and five in the older age group. The median age of the children in the older group, who received the dosage of antibiotics intended for the younger group, was 24 months [IQR 14 to 48]. As drug stock levels were not assessed as part of this study, it is not possible to determine if those children receiving an incorrect dose for their age group were as a result of stock-outs of the correct age-specific blister pack, or that the importance of giving the correct dose for age was not understood by some CHWs. Unfortunately, this was not specifically raised during the CHW FGDs, and it is acknowledged that this is a limitation of the data collected. However, overall, the findings suggest that CHWs are able to prescribe the appropriate dose of antibiotics according to the child’s age.

Interestingly, the study showed that CHWs referred to the flip chart job aid in only 25 % of consultations, and used the sick child reporting form in only 33 %. Although the low utilisation of these tools may be due to the presence of the observer [16], these data could suggest that the CHWs are confident in their ability to recall the diagnostic algorithm and danger signs, given the high level of adherence to the guidelines.

In discussing and analysing data relating to the ability of CHWs, it must be acknowledged that health facility workers with more training may demonstrate a lower level of adherence to guidelines than CHWs for the care of children with cough. For example, one study from Ghana that evaluated the clinical assessments of children presenting to primary health facilities found that the respiratory rate was measured in only 4 % of children presenting with cough, instead of all children as recommended [17].

### Correct use of antibiotics by CHWs

When the data was analysed with a focus on whether the antibiotics prescribed by CHWs were used for the correct purpose, it was found that of the antibiotics given to 202 children in this study, 35 % [95 % CI: 26, 45] were given to children who did not have fast breathing, as assessed by an expert and therefore antibiotics should not have been prescribed. There was a significant difference between the two age groups with regard to the rational use of antibiotics, with antibiotics given to children who were aged 12–59 months more likely to be incorrectly prescribed (45 % [95 % CI: 34, 57] vs 18 % [95 % CI: 11, 30], *p* = <0.01), although it is not clear why this would be the case. The apparent discrepancy between the high number of CHWs able to prescribe antibiotics according to the guidelines and corresponding to their assessment (92 %); and the incorrect use of antibiotics by CHWs (35 %), is in part due to CHWs prescribing antibiotics for children that did not have fast breathing according to their own assessment, but primarily due to their incorrect measurement of the respiratory rate. According to the studies by Cardemil et al. [18] and Miller et al. [19], 24 % and 6 % of antibiotics, respectively, were used irrationally i.e. for the wrong purpose.

In terms of irrational use of antibiotics, where antibiotics were prescribed for the correct purpose i.e. fast breathing pneumonia as assessed by an expert, but not at the correct dosage, 5 % [95 % CI: 2, 11] of all antibiotics given were at the incorrect dose for the child’s age. The difference between the two age groups was not significant (*p* = 0.51). Dosage generally does not appear to have been separately assessed in other research studies, or perhaps is not reported in the literature.

Given that 92 % of children, based on the CHW assessment, were treated as per the guidelines, it is likely that the percentage of antibiotics used irrationally could be substantially reduced with improved tools for the measurement of respiratory rate and an emphasis in training and supervision on the importance of prescribing the correct dose for age. Although it has been recently debated following an article by Druetz et al. [10], the data from this study support the opinion that CHWs are capable of prescribing appropriate treatment in line with guidelines.

### Qualitative findings regarding CHW adherence to guidelines and correct use of antibiotics

The level of knowledge relating to how pneumonia should be treated was fairly good, although the FGDs indicated a higher level of knowledge, than the interviews. This may be simply because the minority of CHWs who contributed in the discussion groups were more vocal because they knew more, or because the group environment helped to stimulate a more detailed discussion.

All of the FGDs correctly mentioned that the medicine for pneumonia is given according to age, with a pink pack for children aged 2–11 months, with one tablet given three times a day (morning, afternoon and evening). For children aged 12–59 months, a green pack is given, with two tablets to be administered three times a day. All groups mentioned that the course of treatment was for 5 days for both age groups. Two of the groups specifically mentioned the name of drug, amoxicillin. One participant in one of the FGDs also mentioned that the CHWs tell “caregivers not to share medicine” (CHW FGD 2).

In terms of the interviews with CHWs, the responses were briefer and less focused. Four of the seven participants who described how they would treat a child with pneumonia mentioned amoxicillin, whilst the others referred simply to medicine given. Four of the participants mentioned that the medicine was for 5 days and two made reference to completing the course of treatment given. Two of the interviews also highlighted the importance of explaining to the caregiver that the child has pneumonia and/or how to give the medicine. Only one participant mentioned the dosage of tablets to be given, although did so incorrectly.

“…child had 1 year 1 month. I gave her amoxicillin 250 a pack of 15 for 5 days”. (CHW IDI 2)

The child was 13 months old, and therefore should have received a pack of 30 tablets for 5 days, allowing for administration of two tablets per dose, three times a day. None of the CHWs in the IDI mentioned the different packs of tablets to be given depending on the age of the child.

In response to the question, ‘What do you think are the things that matter most to your clients regarding the kind of care you provide to them for pneumonia or cough?’, two interview participants specifically referred to the necessity of finishing the course of antibiotics. This was also highlighted by another three participants during the interviews.

“I just encourage the caregivers to give medicine to the patient and finish all the medicine they have been given.” (CHW IDI 8)

### Qualitative data regarding the influences on the prescription and use of antibiotics by CHW

It appears that whilst caregivers are involved in the consultation process, CHWs do not feel influenced when treating children with pneumonia and prescribing medicine, even though some caregivers are perceived to apply pressure to receive medicine.

“When they come, they do not influence us. It’s us to see using our knowledge. When we diagnose, using the knowledge we were taught, then from there we able to know the problem.” (CHW IDI 7)

“I should not base the treatment on the parent’s instructions like our experience today when one parent wanted me to give malaria treatment before I could even attend to the child. We have been trained never to give medicine before checking the child”. (CHW IDI 2)

Whilst CHWs may be unlikely to admit that they are influenced, the data support the quantitative findings. Of those children with normal breathing, as assessed by the CHW, 95 % were not given antibiotics.

The qualitative data, however, does indicate that a large proportion of CHWs feel that caregivers would expect to receive medicine when they attend the CHW and often would pressure the CHW to prescribe medicine or antibiotics.

Whilst there are studies exploring antibiotic use and provider prescribing behaviour, there are very few from low and middle income countries, and even less that are focused on pneumonia or lower respiratory tract infections, and there appears to be none exploring prescribing practices by CHWs. Studies in Peru and Thailand conducted at hospitals and health facilities, relating to treatment for diarrhoea, note that patient expectations can impact antibiotic prescribing behaviour [20, 21]. However, in exploring factors related to the overuse of antimalarials at hospital level in Tanzania, Chandler et al. [22] found that patient satisfaction did not appear to be dependent on prescription of antimalarials. Patients did not explicitly request antimalarials during observed consultations, but there was an expectation that they would be tested and subsequently receive appropriate treatment. In relation to patent medicine vendors in Nigeria, Akuse et al. [23] document a general expectation or preference by caregivers to receive antibiotics as treatment. Chandler et al. [22] also point out that it is likely that social pressures on provider prescribing behaviour may play a greater role in lower level health facilities, which could therefore be further increased for CHWs at community level. In our study, there was a clear desire to receive treatment of some kind, although the data suggest that the emphasis is on treatment that will make the child better, rather than on receiving antibiotics specifically. Furthermore, CHWs did not generally seem to be influenced in our study, despite the perceived social pressure to prescribe medication.

Whilst the care given by the CHW may not be influenced by the caregiver, it appears that this does impact the morale of both CHWs and caregivers. Factors impacting CHWs’ morale should be acknowledged given that their role in Zambia at the time of the study, and other countries, is often voluntary. The FGD that took place with CHWs in Kawambwa appeared to show a greater appreciation for the CHW’s training and knowledge, resulting in less pressure to prescribe medicine. The behaviour change component of the iCCM programme had just begun to be implemented in this district, however it is not possible to definitively attribute this difference in caregiver behaviour to the same communities where the CHWs involved in our study were responsible for providing care.

In our study, the caregivers generally seemed to be satisfied with the care provided by the CHW for children with pneumonia, bearing in mind their apparent discontent if medication was not prescribed. Encouragingly, the reasons for satisfaction expressed by the caregivers were matched by the CHWs’ perceptions of their satisfaction, indicating a good understanding of the community in which the CHWs live and work. Aside from receiving medication and its availability, reasons for satisfaction included availability and accessibility of CHWs, as well as the importance of CHWs’ attitude towards caregivers and quality of care received, similar to reasons noted in a study by Hanson et al. [24] relating to preferences for hospital care in northern Zambia for acute pneumonia in children.

Of note, one participant highlighted that the amoxicillin they receive from the CHW is different to that given by the clinic, which means it is easier for children to swallow, indicating a different formulation. As noted in the background, as part of the iCCM programme in Luapula province, the Pharmaceutical Regulatory Authority in Zambia granted a waiver for the use of dispersible amoxicillin, although it had not been approved for routine use in Zambia.

### Rational use of antibiotics by caregivers

In general, the caregiver who was responsible for administering the drug, was also the same individual that had brought the sick child to the CHW and had received information from the CHW on how to give the antibiotics. All caregivers interviewed stated that they understood the instructions as to how to give the antibiotics, regardless as to how they received them. Although it is not possible to determine exactly what instructions were given to these caregivers from the CHW or another individual, data from the observation of consultations (1490 children) shows that 95 % [95 % CI: 90, 97] of caregivers given amoxicillin received some instructions from the CHW, with 74 % [95 % CI: 62, 83] of those receiving instructions receiving all five key points; number of doses to be given per day, timing, number of tablets per dose, length of treatment course and that all tablets should be taken.

Table 4 displays the results on self-reported administration of antibiotics and pill count. Of the 42 interviews where the age of the child is known, the number of tablets given per dose was generally in line with the iCCM treatment guidelines. Children aged 2–11 months received one tablet per dose and those aged 12–59 months received two tablets as per the guidelines, except for five children who received only one tablet. The median age of these children was 32 months. It is not possible to determine whether this was non-adherence where they only gave one of the two tablets that should have been given, or if they were given the wrong blister pack for their age by the CHW and therefore could only give one tablet per dose. Two children received two or more tablets per dose, but in this instance the child’s age was not specified.

**Table 4:** Self-reported administration of antibiotics by caregivers and pill count

	Age of child			
	2–11 months	12–59 months	Not specified	Total
	*N* = 16	*N* = 25	*N* = 13	*N* = 54^a^
Number of tablets given per dose:				
1 (for children aged 2–11 months)	16 (100 %)	5 (20 %)	3 (23 %)	24 (44 %)
2 (for children aged 12–59 months)	0	20 (80 %)	8 (62 %)	28 (52 %)
> 2	0	0	2 (15 %)	2 (4 %)
Gave amoxicillin 3 times a day	15 (94 %)	23 (92 %)	13 (100 %)	51 (94 %)
Gave amoxicillin morning, noon and night	15 (94 %)	23 (92 %)	12 (92 %)	50 (93 %)
Number of days amoxicillin given:				
2	1 (6 %)	1 (4 %)	0 (0 %)	2 (4 %)
3	16 (%)	6 (24 %)	1 (8 %)	8 (15 %)
4	2 (12 %)	2 (8 %)	1 (8 %)	5 (9 %)
5	11 (69 %)	12 (48 %)	8 (62 %)	31 (57 %)
> 5	1 (6 %)	4 (16 %)	3 (23 %)	8 (15 %)
Number of tablets remaining in the pack:	*N* = 15	*N* = 23	*N* = 12	*N* = 50^b^
0	9 (60 %)	15 (65 %)	9 (75 %)	33 (66 %)
1–6	5 (33 %)	4 (17 %)	2 (17 %)	11 (22 %)
>6	1 (7 %)	4 (17 %)	1 (8 %)	6 (12 %)

^a^Only 54/55 caregivers completed information on timing and number of tablets given

^b^50 caregivers able to present packet of pills

In terms of the timing of doses given, 93 % of caregivers reported that they gave amoxicillin three times a day and at morning, noon and night. However, only 57 % reported that they gave treatment for five consecutive days. 81 % gave treatment for 3 to 5 days, with eight caregivers giving for more than 5 days.

As shown in Table 5, combining the number of days, number of times a day and time when treatment was given into one adherence indicator based on caregiver recall alone indicated that only 54 % of caregivers gave amoxicillin three times a day, at morning, noon and night for 5 days, as required. Adherence increased to 76 %, if those who gave treatment for 3 to 5 days were included (i.e. gave amoxicillin three times a day, at morning, noon and night for 3 to 5 days). A recent Cochrane review [25] found that antibiotics administered for 3 days was as effective as a 5 day course for treatment of non-severe pneumonia in children under five. Our data suggest that a shorter course would potentially improve adherence, considering that caregivers were more likely to be adherent for 3 days than for 5 days. A shorter course of antibiotics could thereby reduce the risk of antibiotic resistance and substantially lower the costs of treatment. However, it must be noted that all the studies in the Cochrane review were from Pakistan and further evidence is needed to support a shorter course of antibiotics for pneumonia in Africa, especially in settings where HIV prevalence is high.

**Table 5:** Indicators of treatment adherence by caregivers

	*n*	*N*	% Adherent [95 % CI]
Self-report indicator:			
Gave amoxicillin, 3 times a day at morning, noon and night for 5 days.	29	54	54 [36 to 64]
Gave amoxicillin, 3 times a day at morning, noon and night for 3, 4 or 5 days	41	54	76 [63 to 85]
Pill-count indicator:			
O tablets	33	50	66 [50 to 79]
Combined indicator:			
0 tablets + gave amoxicillin, 3 times a day at morning, noon and night for 5 days.	23	50	46 [30 to 63]

Looking at pill count alone, of the 50 caregivers who were able to present the packet of pills, 66 % had no tablets remaining. The primary reason that treatment was discontinued, mentioned by nine individuals, was forgetfulness. The second most common explanation was that the patient was better or cured. In the study by Kalyango et al. [26], the same factors were highlighted, with forgetfulness mentioned by 38 % of caregivers and caregivers’ perception of improvement or recovery in the child mentioned by 14 %. It could be concerning that one caregiver specified that the CHW had advised them to stop when the child recovers. However, in the interviews with CHWs, five of the nine participants highlighted the importance of finishing the course of antibiotics and encouraging the caregiver to do so.

A combined indicator for adherence, including both caregiver recall and pill count, indicates that 46 % were fully adherent. Full adherence is defined by giving amoxicillin three times a day, at morning, noon and night for 5 days, based on caregiver recall, and no tablets remaining, as determined by pill count. It is difficult to find data on comparable indicators from studies assessing adherence in higher income countries, however, Kardas and Ratajczyk-Pakalska [27] found that only 23.5 % of patients (aged 18 and over) fully adhered to a 5-day, three times a day, antibiotic treatment regimen for respiratory tract infections in Poland. Adherence was also assessed by pill-count and questionnaire.

High quality pneumonia case management by CHWs is essential for successful treatment outcomes and to minimise any contribution to the development of antibiotic resistance. However, adherence of caregivers to the treatment given is also pivotal for both and to ensure iCCM has the maximum impact in reducing childhood mortality from pneumonia.

Whilst there have been a number of studies on the ability of CHWs to diagnose and treat pneumonia, adherence to courses of antibiotics by caregivers or patients has rarely been explored in a low-income country context (Karin Källander, unpublished observations). However, studies have explored adherence to antimalarials at community level, especially following the change in first line treatment of malaria from chloroquine or sulfadoxine-pyrimethamine to artemisinin combination therapies (ACTs), which required more complex dosing regimens than previous treatments [28–31]. Adherence to a six dose treatment regimen for artemether-lumefrantrine (3 days, twice daily) has been shown to be between 81 and 97 % in some African countries [28, 30, 31], but closer to 40 % in Ethiopia and South Sudan [29]. ACTs are routinely used as part of iCCM in multiple countries.

Adherence has been found to be inversely proportional to dose frequency [32, 33]. However, although children may be treated for more than one disease through iCCM with different dosing regimens, Kalyango et al. [26] found that there was no difference between treatment adherence to antimalarials alone compared with adherence to antimalarials and antibiotics, both 3 day courses of treatment.

### Qualitative data relating to the factors influencing antibiotic use by caregivers

There are multiple factors that may affect the adherence of caregivers to treatment prescribed by a CHW, including knowledge and understanding related to the specific disease, trust and relationship with the CHW, expectation of care and culture or traditional beliefs.

The qualitative data from the focus group discussions with caregivers indicate, perhaps unsurprisingly, that when asked, caregivers all agree that they would follow the instructions given, either in order for their child to be healthy or because they understood the CHW to be appropriately trained.

“The CHWs are well trained and can only give professional advice” (Caregiver FGD 1).

It was implied by one that other caregivers in the community would not finish the course of antibiotics. Also, the caregivers all agreed that they would not share treatment with others.

In terms of caregivers’ knowledge and understanding of pneumonia, one group from one district did seem to have a generally higher level of knowledge, regarding prevention, treatment and prevalence of pneumonia, which may be due to an additional behaviour change communication intervention that had begun to be implemented in certain districts of Luapula province at the time of study. Notably, this group felt that the prevalence of pneumonia had reduced since CHWs had started working in the area, although their perceptions on the prevalence of pneumonia are only likely to reflect their understanding of the disease itself. Whilst knowledge on the causes of pneumonia appeared to be lacking across all the groups, there was an understanding in this group that pneumonia could be prevented, with some suggestions given as to how to prevent pneumonia. In terms of diagnosing pneumonia, the data perhaps imply a level of trust in health workers’ abilities, with caregivers commenting that the health workers would advise as to whether their child needed treatment and that the clinic would not give out medicine without testing the child. It is not clear whether the group was referring solely to health workers based in health facilities specifically, or CHWs. Regarding treatment, the majority of caregivers emphasised that seeking medical help or intervention was the most effective way to treat pneumonia, referring to attendance at the clinic or the hospital. In terms of type of medicine given for pneumonia, there was a large range of comments, but again the group who generally had a higher level of knowledge were more aware of the treatment given, with one individual having a very good understanding, including the number of tablets to be given for children of different ages. However, there were generally mixed responses given on these different topics in the focus group discussions.

Improving caregivers’ understanding of pneumonia and its treatment may improve adherence to treatment and could also impact caregivers’ interactions with CHWs, resulting in greater trust in, recognition of, and increased morale for CHWs. In addition, such BCC interventions may also reduce the likelihood that caregivers will seek medication from elsewhere [34].

## Conclusions

This research provides valuable data on the rational use of antibiotics at community level, by both CHWs and caregivers, through iCCM. From the literature, it appears no other studies have been conducted exploring both the assessment of respiratory rate measurement by CHWs (data presented in a separate manuscript [12]) with evaluation of CHW antibiotic prescribing behaviour, alongside assessment of caregivers’ adherence to treatment in the same setting for iCCM.

The timely identification of children with fast breathing and chest indrawing could prevent the majority of deaths from acute respiratory infections, if appropriate services are available [35]. iCCM improves access to health services for children under five in remote and rural locations, for diseases that have the highest mortality in this age group. Although there is an ongoing debate, this study further supports literature that CHWs providing iCCM are capable of prescribing treatment based on their assessment, in line with guidelines. However, greater accuracy of respiratory rate assessment through improved diagnostic tools would further facilitate the rational use of antibiotics, as well as ensuring the effectiveness of treatment. It is therefore recommended that there is further investment in the development of more user-friendly and refined diagnostic devices for assessing symptoms of pneumonia for use in remote settings in low and middle income countries.

Caregiver adherence to the full 5-day course of antibiotics in this study is less than optimal, despite the use of age-specific blister packs, although evidence suggests that complete adherence could be far higher with a shorter course of treatment [26], also resulting in more rational use of antibiotics. Assessment of effectiveness of a 3-day, compared to a 5-day course of antibiotics for treatment of pneumonia in high HIV settings, such as Africa, would therefore inform decision making on iCCM, helping to maximise adherence and minimise the development of antibiotic resistance.

Furthermore, communities’ perspectives, understanding of a given health issue and their needs should be given sufficient priority and investment in the implementation of health services to ensure interventions can be effectively delivered, for example, through information, education and communication activities.

## Abbreviations

CHW: Community health worker
CI: Confidence interval
CIDA: Canadian International Development Agency
FGD: Focus group discussion
iCCM: Integrated community case management
IDI: In depth interview
IMCI: Integrated management of childhood illness
IQR: Interquartile range
